# Statins as antiepileptogenic drugs: Analyzing the evidence and identifying the most promising statin

**DOI:** 10.1111/epi.17303

**Published:** 2022-06-10

**Authors:** Yousif Hufthy, Mahima Bharadwaj, Shubhi Gupta, Delwar Hussain, Prince Josiah Sajanthan Joseph, Alizah Khan, Jessica King, Pieter Lahorgue, Ovin Jayawardena, Danial Rostami‐Hochaghan, Chloe Smith, Anthony Marson, Nasir Mirza

**Affiliations:** ^1^ School of Medicine University of Liverpool Liverpool UK; ^2^ Department of Pharmacology & Therapeutics Institute of Systems, Molecular and Integrative Biology, University of Liverpool Liverpool UK

**Keywords:** antiepileptogenesis, drug repurposing, epilepsy, epileptogenesis

## Abstract

Many brain insults and injuries are “epileptogenic”: they increase the risk of developing epilepsy. It is desirable to identify treatments that are “antiepileptogenic”: treatments that prevent the development of epilepsy, if administered after the occurrence of an epileptogenic insult. Current antiepileptic drugs are not antiepileptogenic, but evidence of antiepileptogenic efficacy is accumulating for a growing number of other compounds. From among these candidate compounds, statins are deserving of particular attention because statins are reported to be antiepileptogenic in more published studies and in a wider range of brain insults than any other individual or class of compounds. Although many studies report the antiepileptogenic effect of statins, it is unclear how many studies provide evidence that statins exhibit the following two essential features of a clinically viable antiepileptogenic drug: the drug must exert an antiepileptogenic effect even if it is initiated after the epileptogenic brain insult has already occurred, and the antiepileptogenic effect must endure even after the drug has been discontinued. In the current work, we interrogate published preclinical and clinical studies, to determine if statins fulfill these essential requirements. There are eight different statins in clinical use. To enable the clinical use of one of these statins for antiepileptogenesis, its antiepileptogenic effect will have to be established through future time‐ and resource‐intensive clinical trials. Therefore, it is desirable to review the published literature to determine which of the statins emerges as the most promising candidate for antiepileptogenic therapy. Hence, in the current work, we also collate and analyze published data—clinical and pre‐clinical, direct and indirect—that help to answer the question: *Which statin is the most promising candidate to take forward into an antiepileptogenesis clinical trial?*

**Key Points**

- Statins are reported to be antiepileptogenic in more published studies and in a wider range of brain insults than any other compound.
- All available statins have been investigated for potential antiepileptogenic efficacy, and each has shown evidence of benefit.
- Atorvastatin is the statin most commonly used and reported effective in preclinical rodent model studies of antiepileptogenesis.
- Atorvastatin has the most statistically significant association with epilepsy risk reduction in retrospective clinical studies.

## 1 INTRODUCTION

Epilepsy is one of the common chronic neurological diseases globally, and creates a significant burden on individuals and societies. Many brain insults, injuries, symptoms, and diseases are associated with the increased risk of subsequently developing epilepsy, but there are currently no licensed treatments for reducing this risk. Current antiepileptic drugs reduce the risk of experiencing seizures, but do not reduce the risk of developing epilepsy^1^; they are prescribed only once epilepsy has already developed, and are then typically continued long‐term. It is, therefore, desirable to identify treatments that are “antiepileptogenic”: treatments that will prevent the development of epilepsy in the first place, if given after the occurrence of an epileptogenic insult and the onset of epileptogenesis. Epileptogenesis refers to the progressive pathological structural and functional brain changes that follow a brain injury or insult and result in the development of spontaneous recurrent seizures or epilepsy.^2^

Evidence of antiepileptogenic efficacy is accumulating for a growing number of compounds. We searched Medline for studies reporting compounds with antiepileptogenic efficacy, and then manually screened the studies identified. We found that 156 compounds had published reports of antiepileptogenic efficacy (see Table S1). From among these candidate compounds, statins are deserving of particular attention because of the following reasons:

1. Statins are available globally, readily and cheaply.
2. Statins are safe and well tolerated, and they are widely accepted by prescribers and patients. In the UK, for example, atorvastatin is the most commonly prescribed medication (https://opendata.nhsbsa.net/; accessed September 11, 2021).
3. Sizable populations of people at relatively higher risk of developing epilepsy (for example, older people and people with diabetes) can potentially derive cardiovascular and cerebrovascular benefits from statins' lipid‐lowering effect.
4. Statins are reported to be antiepileptogenic in more published studies than any other individual or class of compounds. (Table S1 shows the numbers of published studies that report an antiepileptogenic effect for different compounds. It should be noted that even though statins have the highest number of published reports of antiepileptogenic efficacy, this does not necessarily mean that statins have the highest antiepileptogenic efficacy. The larger number of studies reporting the antiepileptogenic effect of statins, compared to other compounds, could be because statins are favored for research based upon their longstanding and widespread clinical use.)
5. Statins are reported to be antiepileptogenic in a wider range of brain insults (clinical and experimental) than any other individual or class of compounds (Table S1 and Table 1).
6. Statins are the only compounds with preclinical and clinical evidence of potential antiepileptogenic efficacy.
7. Statins are the only compounds with retrospective and prospective clinical evidence of potential antiepileptogenic efficacy.

**TABLE 1:** Patient populations in which statins are reported to reduce the risk of epilepsy/seizures

Population	Point estimate	
Glioblastoma^13^	OR	0.2
Ischemic stroke^23^	OR	0.3
Had early post‐ischemic stroke seizures^17^	OR	0.34
Had radiotherapy for nasopharyngeal carcinoma^22^	HR	0.36
Ischemic stroke^18^	OR	0.41
Ischemic stroke^19^	AHR	0.55
Intracranial haemorrhage^14^	AHR	0.62
Aged ≥66 years^21^	OR	0.64
Age ≥65 years and had coronary revascularization^16^	ARR	0.65
Cerebral cavernous malformations^24^	nr	nr
Ischemic stroke^15^	nr	nr
Ischemic stroke^32^	nr	nr

Abbreviations: AHR, adjusted hazard ratio; ARR, adjusted risk ratio; HR, hazard ratio; nr, not reported; OR, odds ratio.

To be considered a viable antiepileptogenic treatment, a drug must display the following features:

1. The treatment must prevent epilepsy even if it is initiated after the epileptogenic brain insult (for example, stroke or head injury) has already been sustained.
2. The antiepileptogenic effect must endure even after treatment cessation.

If a drug produces a seizure‐preventing effect, but this does not endure after treatment cessation, then it could be an antiseizure drug, rather than an antiepileptogenic drug. If a drug produces an antiepileptogenic effect such that, after a fixed duration of treatment, it can reduce the risk of developing epilepsy in the future, this is better than having to take an antiseizure drug indefinitely/long term, in terms of costs, adverse effects, and quality of life.

Although many studies report the antiepileptogenic effect of statins, it is unclear how many studies provide evidence that statins exert an antiepileptogenic effect even if the statins are initiated after the epileptogenic brain insult has already been sustained, and that the antiepileptogenic effect is sustained even after statins have been discontinued. In the current work, we interrogate published preclinical and clinical studies to answer the following questions:

- Do statins exert an antiepileptogenic effect if they are initiated after the brain insult has already been sustained? How soon after the brain insult must statins be started in order to produce an antiepileptogenic effect? What doses of the statins are needed to exert an antiepileptogenic effect?
- Is the seizure‐preventing effect of statins sustained after discontinuation? How long does statin treatment have to be continued to produce an enduring antiepileptogenic effect?

There are eight different statins in clinical use. Each of the eight statins has been investigated for potential antiepileptogenic efficacy, and each has shown at least some evidence of benefit. To enable the clinical use of one of these statins for antiepileptogenesis, its antiepileptogenic effect will have to be established through future time‐ and resource‐intensive clinical trials. Therefore, it is desirable to review the published literature to determine which of the statins emerges as the most promising candidate for antiepileptogenic therapy. Hence, in the current work, we also aim to collate and analyze published data—clinical and pre‐clinical, direct and indirect—that helps to answer the question: *Which statin is the most promising candidate for antiepileptogenic therapy?* Specifically, we address the following two questions:

- Which statin has the greatest pre‐clinical evidence of antiepileptogenic efficacy?
- Which statin has the greatest clinical evidence of antiepileptogenic efficacy?

To address the preceding questions, we identified the relevant literature, by performing Medline searches tailored to each question, on the July, 15, 2021. Abstracts for the studies found through the Medline search were screened independently by at least two authors to identify relevant studies, which were then read to extract relevant information. Any conflicts between screening authors were resolved by the senior author.

## 2 EVIDENCE FROM PRECLINICAL STUDIES

Three types of rodent models are available for evaluating the antiepileptogenic efficacy of compounds:

1. Kindling models
2. Post‐status epilepticus models
3. Genetic models

All three models have been used to test study the potential antiepileptogenic efficacy of statins. The evidence from each of these three types of models is analyzed separately below.

### 2.1 Kindling models

Kindling is the progressive development of seizures in response to a previously subconvulsant stimulus administered in a repeated and intermittent fashion.^3^ Kindling is a long‐established model of epileptogenesis.^4^ At least seven independent kindling model studies (Table 2) report the antiepileptogenic effect of three different statins (atorvastatin, pitavastatin, and simvastatin). A dose‐dependent ameliorative effect has been reported for each of the statins in at least one study. Atorvastatin was used by a sizable majority of the studies: four of seven. The seven kindling model studies have the following limitations. First, all of the studies have used pentylenetetrazole to conduct kindling. If statins were tested and effective against multiple kindling mechanisms, this would provide stronger support for their potential antiepileptogenic efficacy. Second, statins were given before each pentylenetetrazole injection during kindling acquisition. This means that each statin dose's acute antiseizure effect alone could be sufficient to retard kindling. This experimental design makes it difficult to distinguish drugs truly acting to retard kindling development from those simply masking the expression of kindled seizures.^5^, ^6^, ^7^, ^8^

**TABLE 2:** Kindling rodent model studies that have used statins

Statin	Species	Dose (mg/kg/day)	Before/after	Convulsant	Dose‐dependent effect?
Atorvastatin^39^	Rat	5	Before	Pentylenetetrazole	
Atorvastatin^40^	Mouse	20, 40, 80	Before	Pentylenetetrazole	Y
Atorvastatin^41^	Mouse	10	Before	Pentylenetetrazole	
Atorvastatin^42^	Rat	5	Before	Pentylenetetrazole	
Pitvastatin^43^	Mouse	1 and 4	?	Pentylenetetrazole	Y
Pitvastatin^44^	Mouse	0.5 and 1	Before	Pentylenetetrazole	Y
Simvastatin^45^	Mouse	1, 5 and 10	Before	Pentylenetetrazole	Y

Before/after = statin given before or after convulsant.

### 2.2 Post‐status epilepticus models

In this model, status epilepticus is induced by injection of pharmacological compounds or by electrical stimulation of limbic brain regions; after a latent period, animals exhibit spontaneous recurrent seizures.

Two statins—atorvastatin and simvastatin—have been tested in this model, and both have demonstrated (1) the ability to significantly retard the development of spontaneous recurrent seizures, even though the statins were administered after the brain insult; and (2) sustained benefit after discontinuation of the statins (Table 3).

**TABLE 3:** Post‐status epilepticus model antiepileptogenesis studies that have used statins

Statin	Atorvastatin^10^	Simvastatin^9^	Atorvastatin^11^
Species	Mouse	Rat	Rat
Convulsant	Pilocarpine	Kainic acid	Electrical
Time between insult and initiation of statin (min)	180	30	0
Dose of statin (mg/kg/day)	100	1	10
Duration of statin treatment (days)	14	14	7
Time between cessation of statin and seizure monitoring (days)	1	166	35
Effective?	Yes	Yes	No

From among the post‐status epilepticus model studies, simvastatin has been used in the study^9^ best designed to reflect the potential real‐world clinical application of antiepileptogenic drugs (Table 3), although the interval between status epilepticus and initiation of simvastatin is short at 30 minutes. The study^10^ that reported the antiepileptogenic efficacy of atorvastatin in a post‐status epilepticus model has the following relative limitations: the model does not induce spontaneous recurrent seizures, but rather increases sensitivity to pentylenetetrazole‐induced seizures, and the interval between atorvastatin cessation and seizure monitoring is short at 24 hours. One study found that atorvastatin did not have an antiepileptogenic effect in the post‐status epilepticus rodent model.^11^ Of note, atorvastatin was administered at a lower dose and for a shorter duration in this study, compared to the study that found atorvastatin to have an antiepileptogenic effect in a post‐status epilepticus model.^10^

### 2.3 Genetic models

Atorvastatin (10 mg/kg/day), simvastatin (10 mg/kg/day), and pravastatin (30 mg/kg/day) given orally for 17 consecutive weeks (starting at 45 days of age) significantly reduced the development of absence seizures in adult WAG/Rij rats, a genetic model of absence epilepsy, as demonstrated by electroencephalography (EEG) monitoring 1 month and 5 months after treatment suspension.^12^ This robust study suggests that statins exert an antiepileptogenic effect even if they are initiated when epileptogenesis is already ongoing, and that the seizure‐preventing effect of statins is sustained after discontinuation.

In summary, preclinical studies provide evidence supportive of the following observations:

3 Statins are antiepileptogenic in multiple rodent models of epilepsy—acquired and genetic.
4 Statins are efficacious in preclinical studies designed to identify disease‐modifying (rather than insult‐modifying) effects: statins exert an antiepileptogenic effect even if they are initiated after the brain insult has already been sustained in both the post‐status epilepticus and genetic models of epilepsy.
5 Statins demonstrate sustained antiepileptogenic effect even after they have been discontinued, in both the post‐status epilepticus and genetic models of epilepsy.
6 In terms of the sheer number of pre‐clinical studies reporting an antiepileptogenic effect, atorvastatin comes out on top with nine studies, followed by simvastatin with three studies.

## 3 EVIDENCE FROM CLINICAL STUDIES

### 3.1 Do clinical studies provide evidence that statins are associated with a reduced risk of developing epilepsy?

At least 12 published clinical cohort studies have reported an association between statins and significantly reduced risk of epilepsy/seizures. These clinical cohort studies show that statins are associated with a reduced risk of developing epilepsy not only after an ischemic stroke, but also after brain insults caused by numerous other mechanisms of injury (for example, brain tumour^13^ and intracranial hemorrhage^14^). Different studies reporting an association between statins and reduced risk of epilepsy have been performed in different countries (eg, Brazil,^15^ Canada,^16^ China,^17^ Germany,^13^ Japan,^18^ Taiwan,^14^, ^19^ and the United States^20^, ^21^), in different clinical settings (eg, stroke department,^18^ neurology department,^22^ and neurosurgery department^13^), using different data sources (eg, departmental medical records,^22^ a hospital medical database,^23^ a prospective disease‐specific registry,^24^ a regional administrative health database,^16^ and a national health insurance database^14^), using different study designs (eg, retrospective^17^ and prospective,^15^ cohort^23^ and nested case–control^16^), and using different statistical methods (eg, multivariate Cox regression^22^ and propensity score matching^16^). This makes it less likely that the association between statins and reduced risk of epilepsy identified by these diverse studies can be attributed to a methodological artifact or bias.

The largest of these studies analyzed the data for more than a million individuals from the US Department of Veterans Affairs database, and found that people using statins had an odds ratio of 0.64 (95% confidence interval [CI] = 0.56–0.73) for developing epilepsy.^21^ According to another interesting study, if patients who underwent radiotherapy for nasopharyngeal carcinoma were also taking statins, their hazard ratio for postradiation epilepsy was 0.36 (95% CI = 0.15–0.82).^22^ Another example of a notable finding is that people who started statins after an intracranial hemorrhage had a hazard ratio of 0.62 (95% CI = 0.42–0.90) for developing epilepsy.^14^ Space does not allow a narrative description of all the clinical cohort studies. The studies are summarized in Table 1, and a critical appraisal of these studies can be found in recently published meta‐analyses. At least four different independently published meta‐analyses^25^, ^26^, ^27^, ^28^ of clinical cohort studies have found that statins are associated with a reduced risk of epilepsy. The pooled odds ratio point estimates from the four meta‐analyses range between 0.48 and 0.60.

Whereas 16 clinical studies (cohort studies and meta‐analyses of cohort studies) report that statins are associated with a significant reduction in the risk of developing epilepsy, three clinical cohort studies report that statins are not associated with a significant reduction in the risk of developing epilepsy. It is useful to analyze in detail the minority of studies that report that statins are not associated with a significant reduction in the risk of developing epilepsy, to determine whether methodological issues prevented them from identifying a significant association.

The study of Hseih et al.^29^ did not find a reduced risk of post‐stroke epilepsy in people prescribed statins in hospital. However, the study included people with transient ischemic attacks, who might be at lower risk of developing epilepsy than people with strokes. In addition, any patient given a statin prescription during hospitalization was considered a statin user; it might be that some people used statins too briefly to have a clinical effect. Furthermore, all in‐hospital seizures that occurred during the initial admission were disregarded when people were categorized as having epilepsy or not. However, statin nonusers had significantly higher rates of antiseizure medication use and ventilator‐dependent status epilepticus during the initial admission. Hence, disregarding all in‐hospital seizures that occurred during the initial admission may have led to an underestimation of statins' association with reduction in risk of post‐stroke epilepsy.

The population‐based study of Trivedi et al.^30^ found no association between statin therapy and risk of epilepsy in general and healthy populations. However, the sample size achieved was 14% lower than the required sample size calculated by the authors. Furthermore, the required sample size calculated by the authors might have been underestimated anyway, as it was calculated using an epilepsy population prevalence value (1.5%) that is higher than published values of epilepsy prevalence in the United States and in the patient groups included in the study (0.3% to 1%).

The population‐based study of Molero et al.^31^ applied a within‐individual design to compare the incidence of defined outcomes during periods on statins and periods off statins within each individual; one of these defined outcomes was treatment for seizures. The design of this study means that its results cannot directly inform the question under consideration in the present review. One potential reason is that some who commenced antiseizure medication before starting a statin might continue the antiseizure medication even if their seizures ceased after starting the statin. In addition, if statins are antiepileptogenic, people who used statins for a period but then stopped the statins might have ongoing protection from seizures.

It should be noted that the reduced risk of epilepsy associated with the use of statins after an ischemic stroke could be due to reduced risk of another ischemic stroke, rather than due to an antiepileptogenic effect per se.

### 3.2 Do statins exert an antiepileptogenic effect even if they are initiated after the brain insult has already been sustained?

Some of the clinical cohort studies that have examined the association between statin use and the risk of developing epilepsy after a brain insult have not determined whether the statins were *already* being used by the patients when the brain insult occurred or if the statins were initiated *after* the brain insult occurred. Such studies cannot determine whether statins exert an antiepileptogenic effect even if they are initiated after the brain insult has already been sustained.

Some clinical cohort studies have specifically determined the risk of developing epilepsy for people who initiated statins only after the brain insult had already occurred. One retrospective clinical cohort study found that starting statins *after* a stroke was associated with a significantly reduced risk of developing epilepsy.^19^ Similarly, in another retrospective clinical cohort study, starting statins *after* an intracranial hemorrhage was associated with a reduced risk of developing epilepsy.^14^ In a retrospective clinical cohort study^23^ that reported reduced risk of post‐stroke epilepsy in statin users, >97% of enrolled patients were pre‐stroke statin nonusers. In a prospective clinical cohort study,^15^ people who started statins after a stroke had a reduced risk of developing epilepsy, compared to people who did not start a statin. In another prospective clinical cohort study, patients who started higher dose of statin after a stroke had a reduced risk of developing epilepsy, compared to patients who started a lower dose of a statin after their stroke.^32^

### 3.3 How soon after the brain insult must statins be started in order to produce an antiepileptogenic effect?

The risk of post‐intracranial hemorrhage epilepsy was higher for people who started statins within 2 months of intracranial hemorrhage than for people who started statins within 1 month of intracranial hemorrhage.^14^ In one study, people who started statins within 3 days of a stroke had lower odds of developing epilepsy than people who started statins more than 3 days after a stroke.^15^ On the other hand, a more than 3‐day delay in initiating statin therapy was not associated with a significantly curtailed reduction in the risk of developing post‐stroke epilepsy in another study.^23^ It is notable that statins might still be of benefit after the occurrence of the first seizures: in patients who presented with early‐onset post‐stroke seizures, statin use was associated with a reduced risk of post‐stroke epilepsy.^17^ These observations suggest that starting statins earlier after a brain insult leads to a greater reduction in the risk of developing epilepsy, but the window of opportunity extends beyond the occurrence of the initial seizures.

### 3.4 Is the seizure‐preventing effect of statins sustained after discontinuation?

No published clinical study has been designed specifically to address this question. Only one retrospective clinical cohort study attempted such an analysis,^16^ and reported no benefit for past users of statins, but had only 14 cases in this category, as this was not the study's primary outcome. Some studies provide evidence suggestive of a possible sustained antiepileptogenic effect of statins even after they have been discontinued. In one study, the patient cohort used statins for an average of 4.7 days after an ischemic stroke, but had reduced risk of developing epilepsy (after acute seizures) at more than 2 years of follow‐up.^17^ In another study, people who started but discontinued a statin did not have a higher odds of having epilepsy than people who were still on a statin 1 year after stroke.^15^ These observations suggest the possibility that the antiepileptogenic effect of statins is sustained after they have been discontinued, but this cannot be conclusively determined from the studies that have been performed so far. To conclusively determine if the seizure‐preventing effect of statins is sustained after discontinuation, a randomized controlled clinical trial is needed.

### 3.5 How long does statin treatment have to be continued in order to produce an antiepileptogenic effect?

None of the clinical studies have performed the requisite analysis to specifically address this question. One study found that people who took statins for more than 2 weeks had a significantly lower risk of post‐stroke epilepsy than people who took statins for 2 weeks or less.^23^ This suggests that longer courses of treatment provide greater benefit. It is unlikely that clinical cohort studies can help to quantitate the length of statin treatment needed to produce an antiepileptogenic effect; a clinical trial will be needed to determine this.

### 3.6 What dose of statin produces the greatest antiepileptogenic effect?

Multiple clinical studies report a dose‐dependent relationship between statins and reduction in risk of developing epilepsy.^14^, ^15^, ^16^, ^19^, ^23^ This suggests that the higher the statin dose, the greater the antiepileptogenic effect. In any future clinical trial of a statin for preventing epilepsy, the highest licensed and tolerated dose should be considered.

### 3.7 Which statin has the greatest antiepileptogenic effect?

No clinical study has been designed specifically to compare the antiepileptogenic efficacy of different statins.

Statins that are lipophilic and statins that are hydrophilic have evidence of antiepileptogenic efficacy.^14^, ^19^ In all studies that report a breakdown of cases by statin, the most commonly used statins are atorvastatin and simvastatin.^13^, ^14^, ^15^, ^17^, ^19^, ^23^, ^29^ In the majority of these studies (71%), the most commonly used statin is atorvastatin.^14^, ^17^, ^19^, ^23^, ^29^ In one study^19^ that analyzed multiple individual statins (atorvastatin, fluvastatin, lovastatin, pitavastatin, pravastatin, rosuvastatin, and simvastatin), all statins had a significant association with reduced risk of post‐stroke epilepsy, except for lovastatin and pitavastatin, but these were the two least commonly used statins. Atorvastatin was the most widely used statin in this study and, therefore, had the greatest statistically significant association with epilepsy risk reduction. One study^16^ that performed a dose–response analysis for all individual statins (atorvastatin, lovastatin, cerivastatin, fluvastatin, pravastatin, simvastatin, and rosuvastatin), reported a dose–response relationship for atorvastatin only: a 5% reduction in the risk of epilepsy with every gram of atorvastatin used annually. It may be that the numbers of users of the other statins were insufficient to allow detection of a dose–response relationship, but the data needed to confirm if this is the case are not provided.

In observational clinical cohort studies of statins for epilepsy prevention, the scales are tilted heavily in favor of atorvastatin and simvastatin, as they are used by a much larger number of people, compared to other statins, for hypercholesterolemia and the primary and secondary prevention of cardiovascular and cerebrovascular disease. Hence, atorvastatin and simvastatin have the most clinical studies reporting antiepileptogenic efficacy and the strongest published clinical evidence of antiepileptogenic efficacy. It is unlikely that any future retrospective clinical analysis can overturn the patient‐years numerical advantage favoring atorvastatin and simvastatin. Hence, the greatest retrospective clinical evidence of antiepileptogenic efficacy is and will likely remain for atorvastatin and simvastatin.

## 4 OTHER CONSIDERATIONS WHEN CHOOSING A CANDIDATE DRUG FOR ANTIEPILEPTOGENESIS

### 4.1 Which statin is recommended in the current national guidelines for prevention of cardiovascular disease?

The American College of Cardiology/American Heart Association Task Force on Clinical Practice Guidelines^33^ recommend high‐intensity statin therapy for people with atherosclerotic cardiovascular risk. Their recommendations stipulate that although atorvastatin 80 mg and simvastatin 80 mg are both high intensity, initiation of simvastatin 80 mg or titration to 80 mg is not recommended because of the increased risk of myopathy, including rhabdomyolysis.

In the UK, the National Institute of Health and Care Excellence (NICE) recommends atorvastatin for the primary and secondary prevention of cardiovascular disease (https://cks.nice.org.uk/topics/lipid‐modification‐cvd‐prevention; accessed September 8, 2021). Their recommendation is justified thus. High‐intensity statin therapy is required for primary and secondary prevention of cardiovascular disease. Simvastatin 80 mg and atorvastatin at all doses are considered high intensity. Simvastatin 80 mg is associated with an increased risk of myopathy. NICE concludes that “since equivalent or greater benefits can be obtained from atorvastatin, with a lower risk of myopathy, there is no reason for considering initiating simvastatin 80 mg.”

Hence, atorvastatin, but not simvastatin, can be used at the high‐intensity dose needed for the primary and secondary prevention of cardiovascular disease.

### 4.2 Which statin has the greatest blood–brain barrier permeability?

To determine which of the statins has the greatest antiepileptogenic potential, an additional feature that could be compared is their blood–brain barrier (BBB) permeability. Although a number of chemical features are thought to influence passive diffusion of compounds into the healthy brain (for example, lipophilicity), it is unclear to what degree they influence permeability of drugs into a brain that has sustained an injury. BBB paracellular permeability^34^, ^35^ and the BBB expression of molecular transporters capable of transporting statins^36^, ^37^ increase after epileptogenic brain injury. In addition, chronic administration of statins is thought to upregulate the BBB expression of molecular transporters capable of transporting statins.^38^ Certainly, in multiple brain diseases, both lipophilic and hydrophilic statins have shown evidence of potential disease modification (see preceding text). Therefore, it remains unclear if there is a clinically significant difference in the BBB permeability of different statins after an epileptogenic injury.

## 5 SUMMARY OF EVIDENCE AND FINAL DISCUSSION

Appropriately designed preclinical studies show that statins are antiepileptogenic in animal models. In addition, prospective and retrospective observational clinical cohort studies suggest that the use of statins is associated with a reduced risk of developing epilepsy. A number of observational clinical cohort studies indicate that starting statins even after the brain insult has already occurred reduces the risk of subsequent epilepsy. It is more difficult to determine from observational clinical cohort studies if the antiepileptogenic efficacy of statins persists after the statins have been discontinued. This is because statins are typically continued lifelong in the patient populations included in these observational clinical cohort studies. However, some observations from clinical cohort studies support the possibility that the antiepileptogenic effect of statins is sustained after the statins have been stopped. In preclinical animal models, the antiepileptogenic effect of statins is sustained after the statins have been stopped. Confirmation of this will require appropriately designed interventional clinical trials.

Observational clinical cohort studies suggest that statins should be initiated as soon as possible after the epileptogenic process has been set in motion. However, the antiepileptogenic effect of statins is observed even in patients who have experienced their first seizures. How long does statin treatment have to be continued to produce an antiepileptogenic effect? In animal models, just 2 weeks of treatment has been sufficient. However, longer courses of treatment appear to provide greater benefit. Studies suggest that statins' antiepileptogenic effect is dose‐dependent. Hence, the highest licensed and tolerated dose is likely to have the most antiepileptogenic potential.

There is no preclinical or clinical evidence that any one of the statins has greater antiepileptogenic efficacy than the others. Statins are particularly attractive candidate compounds for an antiepileptogenesis human clinical trial because of the numerous observational clinical cohort studies demonstrating their antiepileptogenic potential. In observational clinical cohort studies of statins for epilepsy prevention, the scales are tilted heavily in favor of atorvastatin and simvastatin, as these agents are used by a much larger number of people, compared to other statins, for hypercholesterolemia and the primary and secondary prevention of cardiovascular and cerebrovascular disease. Hence, atorvastatin and simvastatin have the most clinical studies reporting antiepileptogenic efficacy and the strongest published clinical evidence of antiepileptogenic efficacy. It is unlikely that any future retrospective clinical analysis can overturn the patient‐years numerical advantage favoring atorvastatin and simvastatin. Hence, the greatest retrospective clinical evidence of antiepileptogenic efficacy is and will likely remain for atorvastatin and simvastatin and, hence, one of these two statins should be chosen for future clinical antiepileptogenesis trials.

Based on the evidence reviewed above, the answer to the question *“Which statin is the most promising candidate for antiepileptogenic therapy?”* is, arguably, *atorvastatin*, for the following reasons:

1. Atorvastatin is the most widely used statin in multiple retrospective clinical studies and, therefore, has the most statistically significant association with epilepsy risk reduction of all the statins included in these studies.
2. Atorvastatin is the statin most commonly used and is reported effective in preclinical rodent model studies of antiepileptogenesis.
3. Atorvastatin is favored over simvastatin for the primary and secondary prevention of cardiovascular disease. Therefore, giving people atorvastatin for epilepsy prevention will not compromise their cardiovascular disease–prevention needs.

Although statins are used widely and are generally well tolerated, they are not free from potential adverse effects, like other medicinal compounds. However, unlike novel/experimental compounds, the potential adverse effects of statins (particularly the muscle‐related side effects) are well and widely known, even by members of the public. Therefore, in any future clinical trial, there must be proactive inquiry about such adverse events, and a robust protocol about the necessary actions when they are detected (eg,, dose reduction and increased monitoring).

A large body of evidence built over many years supports considering a randomized controlled clinical trial of a statin for antiepileptogenesis. It is unlikely that performing more preclinical or observational clinical studies will significantly alter the existing case in support of statins. Is it now time for a randomized controlled clinical trial of a statin (likely, atorvastatin) for antiepileptogenesis? If not now, then when?

## AUTHOR CONTRIBUTIONS

Yousif Hufthy: data acquisition; writing, original draft (lead). All authors: data acquisition; writing, review and editing. Nasir Mirza: conceptualization (lead); data curation (lead); writing, review and editing (lead).

## CONFLICT OF INTEREST

None of the authors has any conflict of interest to disclose.

## ETHICAL APPROVAL

We confirm that we have read the Journal's position on issues involved in ethical publication and affirm that this report is consistent with those guidelines.

## Supporting information


Table S1
